# Efficacy and safety of immediate vs. delayed endoscopic retrieval of large or multiple common bile duct stones in high-risk elderly patients: a prospective, randomized comparative study

**DOI:** 10.1007/s10238-025-01712-0

**Published:** 2025-05-29

**Authors:** Omkolsoum Alhaddad, Maha Elsabaawy, Gasser El-Azab, Ahmed Edrees, Mohamed Amer, Mohamed Eissa

**Affiliations:** Hepatology and Gastroenterology Department, National Liver Institute, Menoufia University, Shebeen El-Kom, Menoufia, 32511 Egypt

**Keywords:** Common bile duct stones, Elderly patients, Endoscopic retrograde cholangiopancreatography, Biliary stenting, Extraction

## Abstract

**Introduction:**

Elderly patients with common bile duct (CBD) stones often present with large or multiple stones, making endoscopic retrograde cholangiopancreatography (ERCP)-guided extraction a technically complex and potentially high-risk procedure.

**Aim:**

This study aimed to compare the efficacy and safety of a staged approach—biliary stenting followed by delayed stone extraction—versus immediate stone removal during the initial ERCP in frail elderly patients with large or multiple CBD stones.

**Methods:**

This prospective study included high-risk elderly patients with large or multiple CBD stones, defined as either a single stone ≥ 15 mm in diameter or three or more stones, each ≥ 10 mm in diameter. Participants were randomly assigned to two equal groups: Group A underwent initial biliary stenting with elective stone retrieval after 8–12 weeks, while Group B underwent immediate stone extraction during the first ERCP.

**Results:**

A total of 400 patients were included, with 200 in each group. Baseline characteristics and stone extraction methodologies were comparable between the two groups. In Group A, stone size and number significantly decreased after stenting (mean size: 1.42 ± 0.28 cm before vs. 0.98 ± 0.19 cm after stenting; P < 0.001). The overall stone clearance rate was significantly higher in Group A compared to Group B (94% vs. 80%, P < 0.001). Post-ERCP hospital stay was significantly shorter in Group A (6.41 ± 1.27 days vs. 11.3 ± 1.86 days, P < 0.001). Group B had higher rates of complications, including cholangitis (1% vs. 7%, P < 0.05) and pneumonia (2% vs. 11%, P < 0.05).

**Conclusion:**

In high-risk elderly patients with large or multiple CBD stones, temporary placement of biliary plastic stents followed by elective endoscopic stone removal effectively reduces stone size, simplifies the removal process, enhances stone clearance rates, and decreases ERCP-related complications.

## Introduction

Choledocholithiasis, a common gastrointestinal condition, can lead to serious complications like obstructive jaundice, cholangitis, pancreatitis, and septic shock [1, 2]. Endoscopic sphincterotomy (EST) followed by stone extraction is the standard treatment for common bile duct (CBD) stones [3, 4]. The complexity of stone removal depends on factors such as stone size and number. Large stones often require mechanical lithotripsy for fragmentation before extraction, while multiple stones can prolong and complicate the procedure [5].

When complete stone removal is not possible, a temporary plastic stent is often placed to prevent stone impaction [6]. Previous studies suggest that stent placement can alter stone characteristics and facilitate subsequent removal [7–9]. Some research indicates that initial plastic stent placement without immediate stone extraction can significantly reduce stone size, making them easier to manage in later procedures. [6, 10]

Elderly patients with choledocholithiasis face additional challenges due to increased risks associated with endoscopic retrograde cholangiopancreatography (ERCP), including bleeding, cardiovascular events, and higher mortality rates, especially in those with significant comorbidities [11–13]. While ERCP is less invasive compared to surgical options, the optimal strategy for managing large or multiple CBD stones in high-risk elderly patients remains unclear. Previous studies have demonstrated the efficacy and safety of EST combined with endoscopic papillary large balloon dilation (EPLBD) for large stones in elderly patients [14, 15]. However, data on the best treatment approach for high-risk elderly patients with large or multiple CBD stones are limited.

Given these considerations, our study aimed to evaluate the efficacy and safety of immediate versus delayed endoscopic retrieval of large or multiple CBD stones following temporary stent placement in high-risk elderly patients with chronic diseases.

## Materials and methods

### Study design and patient selection

This prospective, randomized comparative study was conducted at the National Liver Institute, Menoufia University, Egypt, a tertiary referral center specializing in hepatology and gastroenterology with a high volume of ERCP procedures. The study followed the Consolidated Standards of Reporting Trials (CONSORT) guidelines for clinical trial reporting.

The study enrolled high-risk elderly patients diagnosed with large or multiple CBD stones and treated with ERCP between February 2022 and September 2024. Eligible participants were aged 65 or older and had one or more serious chronic conditions, such as cardiovascular disease, cerebrovascular disease, pulmonary disease, diabetes, or chronic liver or kidney disease (Charlson Comorbidity Index (CCI) ≥ 3). [16]

Additional inclusion criteria included the presence of large or multiple CBD stones, defined as either a single stone ≥ 15 mm in diameter or three or more stones, each ≥ 10 mm in diameter, confirmed via computed tomography (CT) or magnetic resonance cholangiopancreatography (MRCP).

Exclusion criteria included inability to provide informed consent, contraindications to ERCP or general anesthesia, anatomical changes due to biliary surgery, benign or malignant strictures in the distal bile duct, intrahepatic bile duct stones, moderate to severe cholangitis [17], and malignancies or other serious conditions with a life expectancy of less than six months.

Eligible patients were randomly assigned to one of two groups: Group A (Stent-first Approach) or Group B (Stone Retrieval-first Approach).

### Study procedures

#### Baseline evaluation

All participants underwent a comprehensive baseline evaluation that included a detailed medical history, a thorough physical examination, laboratory investigations (including liver function tests, complete blood count, and coagulation profile), and imaging studies such as ultrasound or MRCP to assess the size, number, and location of common bile duct (CBD) stones.

### Randomization and blinding

Patients were randomly assigned to either Group A or Group B using a computer-generated randomization list. Allocation was performed in a 1:1 ratio using simple randomization procedures. The randomization list was securely managed and inaccessible to the clinical team enrolling participants, preserving allocation concealment and minimizing the potential for selection bias.

Due to the nature of the interventions, blinding of patients and proceduralists was not feasible. However, outcome assessments—including imaging reviews and complication monitoring—were conducted by independent investigators who were blinded to the treatment groups to minimize observer bias.

#### ERCP procedure

The endoscopic retrograde cholangiopancreatography (ERCP) was performed according to the assigned intervention group. Group A patients had a temporary plastic stent placed to facilitate bile drainage, with elective stone removal scheduled 8–12 weeks later. In Group B, patients underwent immediate stone extraction attempts using conventional techniques such as basket, balloon, or mechanical lithotripsy as needed.

Anesthesia was administered using a standard protocol, tailored to the individual patient. Patients on anticoagulant or antiplatelet medications were managed according to established guidelines. For those unable to discontinue these medications, low molecular-weight heparin was substituted and discontinued as appropriate.

ERCP was performed using a side-view scope Pentax ED-3490 TK (HOYA Corporation, Tokyo, Japan) by experienced endoscopists. Selective cannulation of CBD was achieved using the standard sphincterotome in most patients. In select cases, alternative techniques such as the double-wire technique, pancreatic cannulation with septostomy, or precut sphincterotomy with a needle knife were utilized. Endoscopic sphincterotomy (EST) was performed in all patients. To prevent post-ERCP pancreatitis, we administered rectal indomethacin and maintained adequate hydration as prophylactic measures.

### Stone removal and stent placement

In Group A, after successful cannulation, a single plastic stent (10 Fr, 10 cm) was inserted for drainage, followed by a planned elective ERCP after 8–12 weeks. In Group B, after endoscopic papillary large balloon dilatation (EPLBD) of the papilla, a trial of stone removal was attempted during the first ERCP using either balloon sweeping, Dormia basket, or mechanical lithotripsy. If successful, no stent was placed. However, if stone removal was unsuccessful or incomplete, a plastic stent was inserted. Similar techniques were used for stone extraction and stent insertion in both groups.

### Calculation of stone size and stone index

CBD stone dimensions were measured on radiographs. For patients with multiple stones, all were measured. Additionally, the number of stones was counted. To account for radiographic magnification, the endoscope diameter was used as a reference. Two independent reviewers analyzed the radiographs, and the measurements were averaged. A stone index was calculated to quantify the overall stone burden. This index was determined by summing the diameters (in centimeters) of all the stones present. [8]

### Assessment and follow-up

During the ERCP procedure, technical success was assessed by the ability to achieve stone clearance or proper stent placement. Procedure-related complications, such as pancreatitis, bleeding, or perforation, were documented immediately and monitored throughout the patient's hospitalization. The effectiveness of stone clearance was evaluated based on imaging, laboratory, and clinical improvement.

Patients were scheduled for follow-up visits at 2 weeks, 1 month, and 3 months post-discharge. During these visits, clinical evaluations were conducted, including a review of symptoms, physical examinations, repeat laboratory tests, and imaging studies to monitor for stone recurrence or other biliary complications.

### Outcome measures

The primary outcome of the study was the rate of complete stone clearance, defined as the complete removal of all detectable stones from the common bile duct without the need for additional procedures. Secondary outcomes included the incidence of ERCP-related adverse events during hospitalization, the duration of the procedure, the length of hospital stay, the time from ERCP to discharge, the need for repeat ERCP procedures or alternative biliary interventions due to stone recurrence or complications, and the overall patient morbidity and mortality rates throughout the study period [18, 19]. Post-ERCP cholangitis was defined based on the diagnostic criteria for acute cholangitis outlined in the Tokyo Guidelines 2018 [17], and was considered present when new or worsening clinical signs and symptoms (e.g., fever, abdominal pain, jaundice) developed within 72 h following the ERCP procedure, accompanied by supportive laboratory findings (e.g., elevated white blood cell count, increased bilirubin), in patients who were clinically stable or showed no signs of cholangitis before the procedure. [20]

### Ethical considerations

The study adhered to the principles outlined in the Declaration of Helsinki and received approval from the National Liver Institute's institutional review board (No. 00659). Written informed consent was obtained from all participants before enrollment. For patients unable to provide consent, consent was obtained from their legal guardians.

### Statistical analysis

Data were analyzed using IBM SPSS Statistics, version 22.0 (IBM Corp., Armonk, New York, USA). Categorical data were summarized as frequencies and percentages. The chi-square test was used to investigate associations between categorical variables. When more than 20% of cells had expected counts less than 5, the Monte Carlo correction test or Fisher's exact test was applied.

Continuous data were assessed for normality using the Kolmogorov–Smirnov test. Descriptive statistics for quantitative data included range, mean, standard deviation, and median. For normally distributed quantitative variables, the Student t-test was used to compare two groups. For non-normally distributed quantitative variables, the Mann–Whitney U test was used for group comparisons. Statistical significance was determined at a 5% level.

### Sample size calculation

The sample size was calculated using G*Power 3.1 software to detect a significant difference in stone clearance rates between the two groups. Based on previous literature [6, 9, 11], we anticipated an effect size of 0.15 for this difference with a two-sided α of 0.05 and power of 80%. The estimated required sample size was approximately 175 patients per group (total N = 350). To compensate for potential dropouts or protocol deviations, we increased the sample size to 200 patients per group, resulting in a total of 400 patients enrolled.

## Results

A total of 2378 patients were assessed for eligibility, with 528 identified as high-risk elderly patients with large or multiple CBD stones. Of these, 128 patients were excluded due to various reasons, including refusal to participate (n = 23), being unfit for anesthesia (n = 3), prior biliary surgery (n = 12), moderate or severe cholangitis (n = 41), distal CBD stricture (n = 13), intrahepatic stones (n = 11), and malignancy (n = 25).

**Fig. 1:**
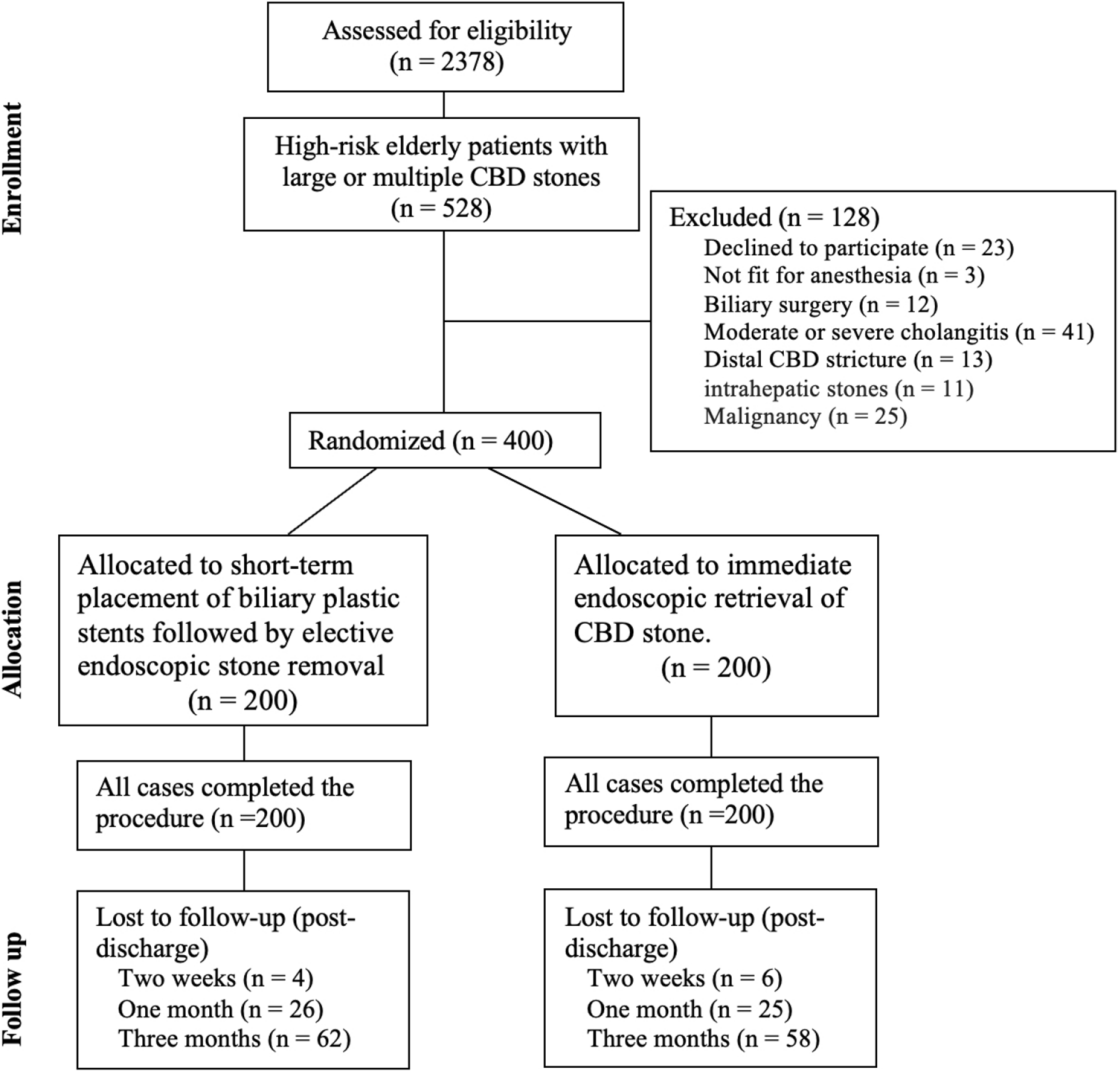
Flow chart of the study

The remaining 400 eligible patients were randomized into two intervention groups: Group A, which underwent short-term placement of biliary plastic stents followed by elective endoscopic stone removal (n = 200), and Group B, which received immediate endoscopic retrieval of CBD stones (n = 200). All cases in both groups completed their respective procedures without dropouts during the intervention phase (Fig. 1).

### Baseline characteristics

The two groups were well-matched regarding baseline characteristics, including age, gender, comorbidities, and stone burden (Table 1). However, Group A had a slightly higher level of total bilirubin (p = 0.029).

**Table 1:** Baseline data of the studied groups

Parameter	Group A (n = 200)	Group B (n = 200)	P value
Sex			
Male	108 (54%)	107 (53.5%)	0.92
Female	92 (46%)	93 (46.5%)	
Age (years)			
Mean ± SD	70.2 ± 3.55	70.4 ± 4.09	0.301
Median (min. – max.)	70 (65–79)	70 (65–79)	
Cholangitis			
Mild cholangitis	56 (28%)	50 (25%)	0.5
Basal bilirubin			
Mean ± SD	8.77 ± 2.45	8.33 ± 2.19	0.029
Median (min. – max.)	9.1 (3.2–17.1)	8 (4.1–13.4)	
CBD diameter (cm)			
Mean ± SD	1.80 ± 0.17	1.88 ± 0.13	0.501
Median (min. – max.)	1.80 (1.40–2)	1.90 (1.50–2)	
Periampullary diverticulum	15 (7.5%)	18 (9%)	0.586
Associated comorbidities			
Diabetes Mellitus	69 (34.5%)	67 (33.5%)	0.833
Respiratory disorders	15 (7.5%)	18 (9%)	0.586
Cardiovascular disorders	86 (43%)	99 (49.5%)	0.192
Chronic renal diseases	18 (9%)	19 (9.5%)	0.863
Cerebrovascular accidents	19 (9.5%)	23 (11.5%)	0.514
Chronic liver disease	78 (39%)	81 (40.5%)	0.759
Charlson Comorbidity Index (CCI)			
Mean ± SD	5.01 ± 1.68	4.85 ± 1.43	0.477
Median (min. – max.)	5 (3–9)	5 (3–8)	

Group A: stent-first approach, Group B: stone retrieval-first approach, min. – max: minimum–maximum, SD: standard deviation, P value: significant if less than 0.5

### Efficacy of stone removal

There were no significant differences between the two groups in terms of cannulation techniques (Table 2). Stone extraction using a balloon was significantly more common among Group A. The successful stone removal rate was significantly higher in the elective ERCP approach in Group A compared to Group B (94% vs. 80%, p < 0.001).

**Table 2:** Techniques of CBD cannulation and stone extraction in the studied groups

Techniques	Group A (n = 200)		Group B (n = 200)	P value
	A1	A2		
Cannulation				
Double wire	12 (6%)	0 (0%)	12 (6%)	1^a^
Precut	19 (9.5%)	0 (0%)	23 (11.5%)	0.514^a^
Standard	169 (84.5%)	200 (100%)	165 (82.5%)	0.59^a^
Stone extraction				
Balloon		165 (82.5%)	137 (68.5%)	0.001^b^
Basket		19 (9.5%)	13 (6.5%)	0.269^b^
Mechanical Lithotripsy		4 (2.0%)	10 (5.0%)	0.103^b^
Failed		12 (6%)	40 (20%)	< 0.001^b^

Group A: stent-first approach, Group B: stone retrieval-first approach, A1: first ERCP in group A, A2: second ERCP in group A, ^a^: between A1 and group B, ^b^: between A2 and group B, P value: significant if less than 0.5

In Group A, 12 patients (6%) failed stone extraction after the second ERCP, necessitating plastic stenting followed by referral to surgery or Spyglass-assisted removal. In Group B, 40 patients (20%) failed stone extraction at the first ERCP. A subsequent attempt was successful in 33 patients, while the remaining cases were referred to surgery or Spyglass-assisted removal.

The procedure time was significantly shorter in the first ERCP and stent insertion in Group A compared to the second ERCP in Group A or the index ERCP in Group B (Table 3).

**Table 3:** ERCP characteristics

	Group A (n = 200)		Group B (n = 200)	P value		
	A1	A2		P^1^	P^2^	P^3^
Size of stones						
Mean ± SD	1.42 ± 0.28	0.98 ± 0.19	1.46 ± 0.35	< 0.001	0.223	< 0.001
Median (min.–max)	1.5 (1–1.9)	1 (0.2–1.5)	1.5 (1–2.3)			
Number of stone						
Mean ± SD	2.05 ± 1.1	1.60 ± 0.74	1.96 ± 1.02	0.001	0.466	0.002
Median (min–max)	2 (1–4)	1 (1–3)	2 (1–4)			
Stone index	2.92 ± 1.97	1.61 ± 1.31	2.87 ± 2.01	< 0.001	0.265	< 0.001
Stone extraction success rate	NA	188 (94%)	160 (80%)	NA	< 0.001	NA
Procedure time (minutes)						
Mean ± SD	28 ± 4.17	43.5 ± 5.91	44.5 ± 7.17	< 0.001	< 0.001	0.141
Median (min.–max)	27.5 (20–35)	44 (31–60)	43 (30–60)			
Post-ERCP hospital stays (days)						
Mean ± SD	6.41 ± 1.27	2.87 ± 0.93	11.3 ± 1.86	< 0.001	< 0.001	< 0.001
Median (min–max)	6 (5–11)	3 (2–7)	11 (8–15)			

ERCP; endoscopic retrograde cholangiopancreatography, Group A: stent-first approach, Group B: stone retrieval-first approach, A1: first ERCP in group A, A2: second ERCP in group A, P^1^ value: between A1 and A2, P^2^ value: between A1 and group B, P^3^value; between A2 and group B, SD: Standard deviation, NA: non-applicable, P value: significant if less than 0.5

### Procedural complications

The overall rate of ERCP-related complications was significantly higher in Group B compared to Group A (24% vs. 16%, p = 0.046). This increase was particularly noticeable in the incidence of post-ERCP cholangitis, which was significantly more frequent in Group B than in Group A (7.5% vs. 1.5%, p = 0.004). Additionally, pneumonia occurred more often in Group B patients (6.5% vs. 2%, p = 0.025). However, there were no significant differences between the groups concerning other complications, such as pancreatitis, bleeding, or cardiovascular events. Notably, no cases of perforation were reported in either group (Table 4). Subgroup analysis revealed that patients with a Charlson Comorbidity Index (CCI) ≥ 5 and those aged over 75 years experienced a significantly higher rate of complications in Group B compared to Group A (p < 0.05).

**Table 4:** ERCP-related adverse events

Adverse events	Group A (n = 200)		Group B (n = 200)	P value^a^
	Subgroups	Total		
Cholangitis	A1: 2 (1%) A2: 1 (0.5%)	3 (1.5%)	15 (7.5%)	0.004
Minor bleeding	A1: 4 (2%) A2: 4 (2%)	8 (4%)	5 (2.5%)	0.398
Acute pancreatitis	A1: 11 (5.5%) A2: 5 (2.5%)	16 (8%)	11 (5.5%)	0.319
Perforation	A1: 0 (0%) A2: 0 (0%)	0 (0%)	0 (0%)	NA
Atrial Fibrillation	A1: 0 (0%) A2: 1 (0.5%)	1 (0.5%)	4 (2%)	0.177
Pneumonia	A1: 4 (2%) A2: 0 (0%)	4 (2%)	13 (6.5%)	0.025
**Total**	**A1: 21 (10.5%)** **A2: 11 (5.5%)**	**32 (16%)**	**48 (24%)**	**0.046**

Group A: stent-first approach, Group B: stone retrieval-first approach, A1: first ERCP in group A, A2: second ERCP in group A, NA: non-applicable, ^a^: between total number of Group A and Group B, P value: significant if less than 0.5

### Hospital stay and mortality

The average length of hospital stay was significantly longer in Group B. No deaths were recorded in Group A, while six patients died in Group B following ICU admission for sepsis and respiratory failure (p = 0.03).

### Comparison of first and second ERCP procedures

In Group A, we compared the bile duct stone diameter, ERCP procedure time, adverse event rate, and length of hospital stay for the first and second ERCP procedures.

- **Stone Size:** Following stent drainage, the maximum stone size and stone index decreased in 158 patients (79%). The diameter of CBD stones was significantly reduced (1.42 ± 0.28 cm vs. 0.98 ± 0.19 cm, p < 0.001).
- **Procedure Time:** The second ERCP took significantly longer time than the first (43.5 ± 5.91 min vs. 28 ± 4.17 min, p < 0.001).
- **Hospital Stay:** The average length of hospital stay was significantly longer for the first ERCP (6.41 ± 1.27 days vs. 2.87 ± 0.93 days, p < 0.001).
- **Adverse Events:** There was no significant difference in the overall ERCP-related adverse event rate (10.5% vs. 5.5%, p = 0.065) between the two procedures.

### Medical costs

Despite the shorter hospital stay, estimated costs were higher in Group A. The average cost for ERCP sessions was approximately 25,000 Egyptian pounds in Group A, compared to 15,000 Egyptian pounds in Group B.

The total cost per patient was higher in Group A (35,000 Egyptian pounds or 700 US dollars) compared to Group B (27,000 Egyptian pounds or 540 US dollars).

## Discussion

Diseases of the pancreaticobiliary tract are common among elderly individuals, with common bile duct stones and malignancies accounting for over 70% of jaundice cases in patients older than 75 years [21]. In this age group, biliary tract disease is the leading cause of abdominal surgery, which carries a high risk of complications and mortality [12]. Endoscopic retrograde cholangiopancreatography (ERCP) reduces the need for urgent surgery and lowers morbidity in these patients [22]. With the increasing elderly population, the number of ERCP procedures has risen globally, but older patients are considered at higher risk for ERCP-related complications because of multiple comorbidities. [13]

Large common bile duct (CBD) stones (≥ 1.5 cm) and multiple stones present challenges for endoscopic removal, decreasing the success rate of traditional methods like endoscopic sphincterotomy (EST) combined with basket or balloon techniques [10]. Other techniques such as mechanical lithotripsy or EST combined with endoscopic papillary large balloon dilation (EPLBD) can improve success rates, though they increase the procedure's complexity and duration, and raise the risk of complications, especially in elderly patients with comorbid conditions [11]. For difficult-to-remove stones, current guidelines recommend temporary stent placement for bile duct drainage to prevent complications like stone impaction and cholangitis [7, 10]. Elective endoscopic stone removal following short-term stenting is a commonly adopted strategy.

Previous studies have demonstrated that combining EST with EPLBD is a safe and effective approach for removing large CBD stones in elderly patients [14, 15]. However, there is limited research on whether immediate endoscopic stone removal or a strategy involving short-term bile duct stent placement followed by elective stone removal is safer and more effective for this population, especially those with chronic diseases and poor overall health.

Our study evaluated the efficacy and safety of temporary bile duct stenting followed by elective stone removal compared to immediate stone removal in high-risk elderly patients with large or multiple CBD stones. Results showed a higher stone clearance rate and a lower incidence of ERCP-related adverse events in the stent drainage group compared to the immediate removal group.

For treatment of choledocholithiasis, EST with balloon or basket extraction remains the standard, with a clearance rate of 80–92.3%. [23–26]. For large or multiple stones, elective removal after short-term stent placement shows a more than 90% clearance rate. [6, 27, 28]

Han J. and colleagues reported that endoscopic stone removal was successful in 92.8% of patients (26 out of 28) following plastic stenting and the use of ursodeoxycholic acid [29]. However, the European Society of Gastrointestinal Endoscopy (ESGE) advises against using ursodeoxycholic acid or other choleretic agents for the treatment of common bile duct stones (CBDSs) or for preventing their recurrence after endoscopic clearance [7]. Meng K. and his team found that the stone clearance rate in the group undergoing elective stone removal was higher than in the immediate stone removal group (89.5% vs. 75.3%, P = 0.049) [11]. Our study showed similar results, with success rates of 80% in the immediate removal group and 94% in the group receiving stent drainage followed by elective stone removal. We believe this difference is due to the reduction in size and hardness of the CBD stones after short-term stent placement.

Our study demonstrated that after short-term stenting, the average size of CBD stones decreased significantly from 1.42 to 0.98 cm. Meng K. et al. observed a similar reduction, with the main CBD stone diameter decreasing from 1.95 to 1.59 cm. They also noted that in one patient, no stones were detected during the second ERCP, suggesting the possibility of spontaneous stone removal due to fragmentation from stent placement [11]. Similar findings have been reported in previous studies [9, 27, 30]. While short-term biliary stent placement can reduce the size and number of CBD stones, the precise mechanism remains unclear. It is hypothesized that the friction between the stent and the stones caused by breathing and intestinal movements may lead to stone fragmentation. Additionally, changes in bile composition due to the reflux of duodenal contents (intestinal fluid or gas) following stent placement could also play a role. [6, 29]

In our cohort, post-stenting downsizing was observed in 79% of patients in Group A. However, in cases where stone removal remained difficult, most stones showed minimal or no size reduction, and nearly all involved a single large stone > 1.6 cm. These findings suggest that initial stone size may serve as a useful predictor of response to stenting and could assist in selecting patients most likely to benefit from a stent-first strategy. Although stone hardness and composition were not quantitatively evaluated in this study, they are likely to influence outcomes. Future research should incorporate radiologic parameters, such as CT attenuation values, to better assess stone density and composition. Developing predictive models that integrate stone size, number, and radiologic density may enhance individualized decision-making in the endoscopic management of complex CBD stones.

In our study, we used plastic stents for all patients undergoing biliary stenting. Meng et al. used double pigtail stents in their study and reported similar results to ours in terms of stone size reduction and stone clearance rates [11]. However, there is limited data available on the most suitable types of biliary plastic stents for short-term placement and the optimal duration for which they should remain in place. Additionally, there is little information on whether placing one or more biliary stents is more advantageous.

The only available retrospective study on this matter included 64 elderly patients (aged 65 and older) with large (≥ 20 mm) or multiple (≥ 3) common bile duct (CBD) stones. These patients received either a single or double plastic stent during the initial ERCP. Approximately three months later, a second ERCP was conducted to attempt stone removal using standard techniques. This study found that double plastic biliary stenting (7 or 8.5 Fr) was more effective than single stenting (8.5 Fr) in maintaining stent patency over three months. However, both approaches were equally effective in reducing the size and number of stones, with no significant differences in complication rates between the two groups. [9]

The overall incidence of ERCP-related adverse events was higher in Group B compared to Group A. Specifically, the incidence of cholangitis was 7.5% in Group B, significantly higher than the 2.5% observed in Group A. This difference might be due to the more frequent use of balloon/basket or mechanical lithotripsy for stone extraction in Group B, which increases the volume of contrast dye used and prolongs the procedure time, thereby raising the risk of biliary infection. Additionally, pneumonia was more common in Group B, occurring in 6.5% of patients compared to 2% in Group A. The higher rate of pneumonia in Group B could be attributed to the longer procedure times, which increased the risk of aspiration during recovery, leading to higher rates of pneumonia and sepsis, and a higher mortality rate (3% vs. 0%).

Our subgroup analysis suggests that the stent-first approach may be more appropriate for patients with significant comorbidities (CCI ≥ 5) or those aged over 75 years. These high-risk patients exhibited higher rates of complications when managed with the stone retrieval-first approach. Although the stent-first strategy entails higher initial costs due to the requirement for two ERCP sessions, it appears to offer superior safety outcomes in frail or vulnerable populations. These findings underscore the need to individualize ERCP strategies based on patient risk profiles, prioritizing safety over cost in those at increased risk of adverse events.

Previous studies have shown that elderly patients are at a higher risk of cardiovascular events following ERCP, and a procedure time exceeding 30 min significantly elevates the risk of myocardial injury in this population [31]. However, in our study, although the ERCP procedure duration was over 30 min for Group B and during the second ERCP for Group A, the only cardiovascular event reported was atrial fibrillation. There was no significant difference in the incidence of atrial fibrillation between the two groups. When comparing the incidence of adverse events between the two ERCP procedures in Group A, there was no significant difference in the rate of ERCP-related complications, even though the procedure time was longer during the second ERCP.

Based on these findings, it seems reasonable to conclude that stent drainage followed by elective stone removal is a safe approach and does not increase the overall incidence of ERCP-related adverse events, even if two ERCP procedures are required for each patient.

This study has several limitations. First, being a single-center study, the results may not be generalizable to other clinical settings. Second, the study did not investigate which type or number of biliary plastic stents is most effective for short-term placement. Third, the optimal interval between stent insertion and elective ERCP was not determined. Fourth, stone hardness was not assessed in this study, although it may aid in predicting which stones are more likely to significantly reduce in size following stenting. Lastly, although our inclusion criteria primarily focused on the size and number of stones, we acknowledge that anatomical variations, particularly in the configuration of the distal bile duct, may also influence procedural complexity. While these parameters were not systematically evaluated in the present study, they warrant consideration in future research aimed at refining endoscopic risk stratification.

Nevertheless, to our knowledge, this is the first prospective study with a large patient cohort to evaluate the efficacy of two different approaches for retrieving large or multiple CBD stones in elderly patients. Additionally, it offers a comparison of the financial costs associated with each treatment strategy.

In conclusion, our study demonstrates that in high-risk elderly patients with large or multiple CBD stones, short-term biliary plastic stent placement followed by elective endoscopic stone removal effectively reduces stone size and simplifies the extraction process. This approach significantly improves the rate of complete stone clearance and reduces ERCP-related complications, making it particularly beneficial for elderly patients with chronic illnesses and compromised overall health.

## Data Availability

No datasets were generated or analysed during the current study.
